# Induction of integration-free human-induced pluripotent stem cells under serum- and feeder-free conditions

**DOI:** 10.1007/s11626-019-00412-w

**Published:** 2019-11-25

**Authors:** Atsuko Hamada, Eri Akagi, Sachiko Yamasaki, Hirotaka Nakatao, Fumitaka Obayashi, Manami Ohtaka, Ken Nishimura, Mahito Nakanishi, Shigeaki Toratani, Tetsuji Okamoto

**Affiliations:** 1 Department of Oral and Maxillofacial Surgery, Hiroshima University Hospital, 1-2-3, Kasumi, Minami-ku, Hiroshima-City, 734-8553 Japan; 2 Department of Oral Maxillofacial Surgery, Hiroshima Red Cross Hospital & Atomic-bomb Survivors Hospital, Hiroshima, Japan; 3 TOKIWA-Bio, Inc., Tsukuba, Ibaraki, Japan; 4 Biotechnology Research Institute for Drug Discovery, National Institute of Advanced Industrial Science and Technology (AIST), Tsukuba, Ibaraki, Japan; 5 Laboratory of Gene Regulation, Faculty of Medicine, University of Tsukuba, Tsukuba, Ibaraki, Japan; 6 Department of Molecular Oral Medicine and Maxillofacial Surgery, Division of Applied Life Science, Graduate Institute of Biomedical and Health Science, Hiroshima University, Hiroshima, Japan

**Keywords:** Human-induced pluripotent stem cells, Peripheral blood mononuclear cells, Reprogramming efficiency, hESF9 serum-free defined media

## Abstract

Human-induced pluripotent stem cells (hiPSCs) have shown great potential toward practical and scientific applications. We previously reported the generation of human dental pulp stem cells using non-integrating replication-defective Sendai virus (SeVdp) vector in feeder-free culture with serum-free medium hESF9. This study describes the generation of hiPSCs from peripheral blood mononuclear cells to increase the donor population, while reducing biopsy invasiveness. From 6-d-old primary culture of peripheral blood mononuclear cells (PBMCs) with IL-2, hiPSCs were established using SeVdp(KOSM)302L with recombinant Laminin-511 E8 fragments under serum-free condition. The established PBMC-derived hiPSCs showed pluripotency and differentiation ability both *in vivo* and *in vitro*. In addition, we evaluated microarray data from PBMC- and dental pulp–derived hiPSCs. These hiPSCs will be beneficial for characterizing the molecular mechanisms of cellular differentiation and may provide useful substrates for developing cellular therapeutics.

## Introduction

Human-induced pluripotent stem cells (hiPSCs) (Takahashi and Yamanaka 2006) research holds tremendous potential for regenerative medicine, drug discovery, and disease modeling with the added advantage of circumventing the ethical concerns regarding the use of pluripotent cells derived from embryos. Ever since Yamanaka and colleagues established a cell bank of donor-derived hiPSCs, rather than making them for each patient (Andrews *et al.*^2014^), the demand for high-quality hiPSCs has risen significantly.

hiPSCs are typically induced and cultured from feeder cells in serum-containing medium in conventional culture systems. Murine-derived feeder cells are widely used to maintain the pluripotency of hiPSCs, and human-derived feeder cells are also used. However, these cells are unsuitable for stem cell maintenance (Price 2017) as the feeder cells are cultured in medium supplemented with fetal bovine serum or proprietary serum replacements. The use of culture medium containing undefined or unknown components has limited the understanding of development and cellular differentiation (Nims and Harbell 2017).

DNA-integrative retroviral and lentiviral vectors, first described by Yamanaka (Takahashi and Yamanaka 2006), have been used widely in cell reprogramming because they stably express transgenes, resulting from the chromosomal insertion of the vector (Takahashi *et al.*^2007^) (Yu *et al.*^2007^) (Lowry *et al.*^2008^). However, the therapeutic potential of hiPSCs is complicated by the potential risks posed by continuous expression of transgenes and by random genome integration of viral vectors. Recently, a number of procedures have been introduced to generate genetically non-integrative or unmodified hiPSCs. These approaches involve chemicals or plasmid, episomal, or viral vectors (Kaji *et al.*^2009^) (Woltjen *et al.*^2009^) (Zhou *et al.*^2009^) (Jia *et al.*^2010^) (Warren *et al.*^2010^) (Yu *et al.*^2011^); however, they show extremely low efficiency in generating iPSCs.

To prevent the risk of contaminating hiPSCs with unknown viruses, unknown substances, and unpredictable genome insertions, as well as to standardize a culture method under defined conditions, we previously developed and reported culture systems capable of maintaining both the undifferentiated status and pluripotency of embryonic stem cells (ESCs) and iPSCs without using serum or feeder cells or retroviruses from dental pulp cells (DPCs) (Yamasaki *et al.*^2016^), eliminating the risk of activating nearby oncogenes by gene insertions. To further simplify the generation of hiPSCs and to increase the donor population, we report here the generation of hiPSCs using peripheral blood mononuclear cells (PBMCs) from seven healthy adult donors of various ages as sources of hiPSCs. This procedure was carried out without invasive and painful biopsies.

Based on our preliminary experimental results, we performed hiPSC generation from PBMCs using Laminin-E8 in completely serum-free culture conditions after 6 d of primary culture. The system can maintain the pluripotency of the cells and retain their potential to differentiate into all three embryonic germ layers, as well as DPC-derived hiPSCs (Yamasaki *et al.*^2016^). We also compared microarray data from PBMC- and DPC-derived hiPSCs. This culture system will be beneficial for evaluating the molecular mechanisms under defined conditions. Furthermore, the combination of this system and the generation of patient-derived iPSCs will contribute to attaining new knowledge to overcome various disorders.

## Materials and Methods

### **Cell lines**

This study was carried out using dental pulp cell (DPC)–derived hiPSCs (DPC-hiPSCs) and PBMC-derived hiPSCs (PBMC-hiPSCs) (Table 1). The generation of DPC-hiPSCs was previously reported (Yamasaki *et al.*^2014^; Yamasaki *et al.*^2016^). Briefly, human DPCs were cultured in a gelatin (Millipore, Billerica, MA)-coated 12-well plate at a density of 1 × 10^5^ cells/well in RD6F serum-free medium (Myoken *et al.*^1989^), and then transfected with the Sandai virus (SeVdp)(KOSM) vector (Nishimura *et al.*^2011^) at a multiplicity of infection (MOI) of 6 once at room temperature for 2 h and then incubated at 37°C overnight in a humid atmosphere of 95% air/5% CO_2_ in RD6F medium. The transfected cells were trypsinized and seeded onto a fibronectin (2 μg/cm^2^) (Sigma-Aldrich, St. Louis, MO)-coated 6-well plate at a density of 1.0 × 10^4^ cells/well in hESF9 medium (Furue *et al.*^2008^) (Ohnuma *et al.*^2014^) (Yamasaki *et al.*^2013^) at 38°C in a humid atmosphere of 95% air/5% CO_2_. The medium was changed every other day. At approximately 14 d after transduction, hiPSC colonies were picked based on human ESC-like colony in morphology. The picked colonies were mechanically dissociated into small clumps and subsequently expanded and maintained on fibronectin-coated dishes in hESF9 with TGF-β1 (2 ng/mL) or activin A (10 ng/mL) at 37°C, 95% air/5% CO_2_ as described previously (Hayashi *et al.*^2010^) (Yamasaki *et al.*^2013^).

**Table 1.** The list of hiPSC cell lines. Hamada et al.

Serial number	Cell type	Sex	Name of cell line
No. 1	DP	Female	WT 1-DP-hiPSC
No. 2	DP	Female	WT 2-DP-hiPSC
No. 3	PBMC	Male	WT 3-PBMC-hiPSC
No. 4	PBMC	Male	WT 4-PBMC-hiPSC
No. 5	PBMC	Female	WT 5-PBMC-hiPSC
No. 6	PBMC	Male	WT 6-PBMC-hiPSC
No. 7	PBMC	Male	WT 7-PBMC-hiPSC
No. 8	PBMC	Male	WT 8-PBMC-hiPSC
No. 9	PBMC	Female	WT 9-PBMC-hiPSC

### **Isolation and culture of PBMCs in serum-free medium**

We obtained human blood samples from healthy volunteers at Hiroshima University Hospital for the use of the blood to generate hiPSCs in accordance with approved guidelines. PBMCs were prepared by density gradient centrifugation in a Histopaque 1077 (Sigma-Aldrich) and cultured in RD6F serum-free medium supplemented with IL-2 (CELEUK, Takeda Pharmaceutical Co., Osaka, Japan) (Sato *et al.*^1987^; Okamoto *et al.*^1996^) for 0–6 d at 37°C in a humidified atmosphere of 95% air/5% CO_2_.

### **Induction of PBMC-hiPSCs with SeVdp(KOSM)302L**

**Figure 1.**
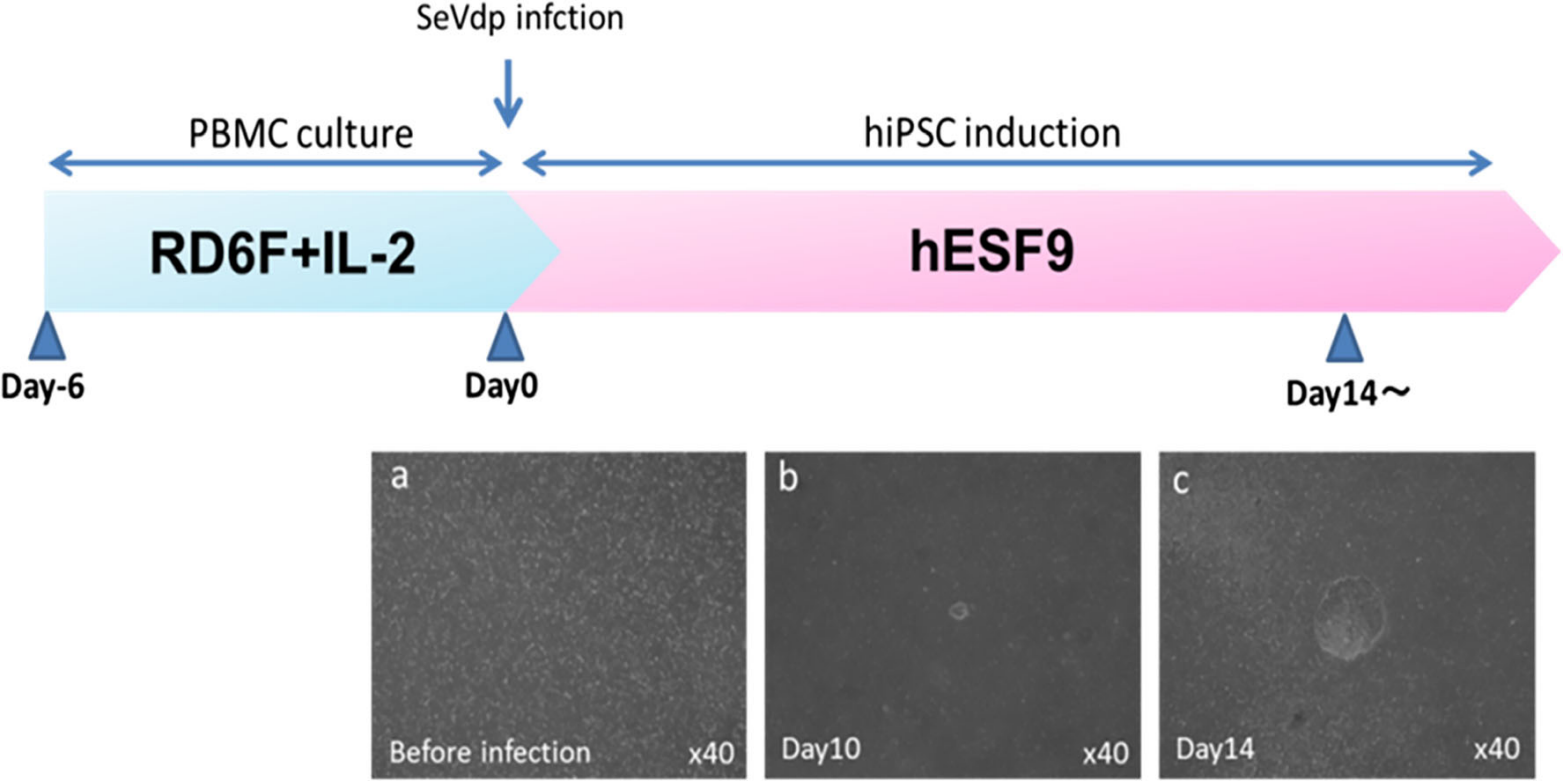
Procedure for hiPSC induction under serum-, feeder-, and integration-free conditions. Time schedule of hiPSC induction. (*a*) Phase contrast images of WT4-PBMCs on Day 0. (*b*, *c*) Phase contrast images of WT4-PBMC on Day 10 (*b*) and Day 14 (*c*) after SeVdp transfection. ESC-like colonies were detected approximately 14 d after transduction. Hamada et al.

An outline of experimental procedure for inducing PBMC-hiPSCs with SeVdp(KOSM)302L (Nishimura *et al.*^2017^) is shown in Fig. 1. WT-PBMCs were transfected at a density of 1 × 10^5^ cells with SeVdp(KOSM)302L, which does not integrate into the host genome, at an MOI of 6 for 2 h at 32°C in RD6F medium in a 48-well plate (BD Biosciences, Falcon®, Franklin Lakes, NJ) in a humid atmosphere of 95% air/5% CO_2_. The transfected cells were collected by centrifugation at 200×*g* for 5 min and then seeded onto a Laminin-E8 (0.5 μg/cm^2^) (Nippi, Tokyo, Japan)-coated 6-well plate (BD Biosciences, Falcon®), in hESF9 medium at 38°C as described above. The medium was exchanged every other day. At approximately 14 d after transduction, detected colonies were mechanically picked with a 200-μL pipette and further cultured in a Laminin-E8-coated 4-well plate (Thermo Fisher Scientific, Waltham, MA) in hEFS9 with TGF-b1 or activin A. The PBMC-hiPSCs were passaged every 5–7 d by mechanical procedure as described above. The reprograming efficiency was determined as the alkaline phosphatase (ALP) positive colonies per total number of infected cells. For the control culture, transfected PBMCs were seeded onto mitomycin-C-treated mouse embryonic fibroblasts (MEFs) (Millipore) as feeder layer cells under KnockOut™ Serum Replacement (KSR) (Thermo Fisher Scientific)-based culture conditions.

### **ALP staining**

ALP staining was performed using a Fast Red substrate kit (Nichirei Biosciences, Inc., Tokyo, Japan) according to the manufacturer’s protocol. Images of the dish were acquired using LUMIX (Panasonic, Osaka, Japan) and positive areas were detected using ImageJ software (National Institutes of Health, Bethesda, MD).

### ***In vitro*and*in vivo*differentiation of PBMC-hiPSCs**

The *in vitro* and *in vivo* differentiation of PBMC-hiPSCs was performed as described previously (Yamasaki *et al.*^2014^). Briefly, to confirm the *in vitro* differentiation capacity of PBMC-hiPSCs, an embryoid body assay was performed. Undifferentiated PBMC-hiPSCs were cultured in hESF6 without FGF2, heparin, and TGF-b1 or activin A in low-attachment 96-well plates (Sumitomo Bakelite Co., Ltd., Tokyo, Japan) for 4–5 d. Then, 3–5 embryoid bodies (EBs) were transferred to gelatin-coated 35-mm dishes and further cultured for another 21 d in hESF6. The medium was changed every 3–5 d. The cells were fixed and stained with the antibodies shown in Table 1. For *in vivo* studies, PBMC-hiPSCs were injected into the dorsal flank of SCID (CB17/Icr-Prkdcscid/CrlCrlj) mice (1 × 10^6^ cells/100 μL of the cell suspension). Approximately 10 wk after injection, the teratoma were surgically dissected, fixed using phosphate-buffered saline solution containing 4% formaldehyde, and embedded in paraffin. Then each section was stained with hematoxylin/eosin and Alcian blue/PAS. The histological findings were evaluated using a Nikon ECLIPSE E800 microscope (Tokyo, Japan) and photographed using a Leica DC500 camera (Leica Microsystems AG, Wetzlar, Germany).

### **RNA isolation and reverse transcription gene expression**

Total RNA was isolated from PBMC-hiPSCs using TRIzol RNA Isolation Reagent (Thermo Fisher Scientific) according to the manufacturer’s protocol. The cDNAs were synthesized from 1 μg of total RNA using high-capacity RNA to cDNA master mix (Applied Biosystems, Foster City, CA). RT-PCR was performed with KOD-FX neo (Toyobo, Osaka, Japan) using primers (Table 2). The PCR products were size-fractionated by 1.5% agarose gel electrophoresis and imaged with the ChemiDoc Touch Imaging System (Bio-Rad, Hercules, CA). RT-qPCR was carried out on an AiraMx Real-time PCR system (Agilent Technologies, Santa Clara, CA) using FastStart Universal Probe Master (ROX) (Roche Diagnostic K.K., Basel, Switzerland). Each 10-μL reaction contained 5.0 μL of FastStart Universal Probe Master (ROX); 0.1 μL of each Universal ProbeLibrary Probe (Roche) (Table 3), 0.2 μL of each primer (25 μM) (Roche); 0.5 μL of cDNA template (~ 25 ng/μL); and 4.0 μL of RNase-free dH_2_O. The cycle program for product amplification was 1 cycle of 95°C for 10 min (hot-start activation), followed by 40 cycles of 95°C for 30 s (denaturation), 55°C for 1 min (annealing), and 72°C for 1 min (extension).

**Table 2:** The List of primer sequences for RT-PCR

Gene Name	Primer sequence	Product size (bp)	Reference
Oct3/4	5’-GACAGGGGGAGGGGAGGAGCTAGG-3′ 5’-CTTCCCTCCAACCAGTTGCCCCAAAC-3′	144	Yamanaka S. et al.
Nanog	5’-CAGCCCCGATTCTTCCACCAGTCCC-3′ 5’-CGGAAGATTCCCAGTCGGGTTCACC-3’	366	Yamanaka S. et al.
Sox2	5’-GGGAAATGGGAGGGTGCAAAAGAGG-3′ 5’-TTGCGTGAGTGTGGATGGGATTGGT-3’	151	Yamanaka S. et al.
Rex1	5’-CAGATCCTAAACAGCTCGCAGAAT-3′ 5’-GCGTACGCAAATTAAAGTCCAGA-3’	306	Yamanaka S. et al.
SeVdp (NP)	5’-AGACCCTAAGAGGACGAAGA-3′ 5’-ACTCCCATGGCGTAACTCCATAGTG-3’	700	Nakanishi K. et al.
GAPDH	5’-TGATGACATCAAGAAGGTGGTGAAG-3′ 5’-TCCTTGGAGGCCATGTGGCCAT-3’	240	Furue MK. et al.

### **Immunocytochemistry**

To examine the pluripotency of the PBMC-hiPSCs, immunocytochemistry was performed as described previously (Yamasaki *et al.*^2014^). Briefly, the cells fixed with 4% paraformaldehyde were stained with antibodies against Oct4 (MAB4401, mouse monoclonal, 1/200, Millipore), Tra-1-60 (09-0010, mouse monoclonal, 1/200, Stemgent®, Cambridge, MA), and SSEA-4 (MC 813-70, mouse monoclonal, 1/100, R&D Systems Minneapolis, MN), and differentiated cells were stained with antibodies against βIII-tubulin (MAB3408/1637, mouse monoclonal, 1/300, Chemicon, Burlington, MA), α-smooth muscle actin (N1584, mouse monoclonal, pre-diluted, DAKO Cytomation, Glostrup, Denmark), and α-fetoprotein (MAB1368, mouse monoclonal, 1/100, R&D Systems) (Table 1). These primary antibodies were visualized with Alexa Fluor® 488-conjugated goat anti-mouse IgG (A11001, 1/300, Invitrogen, Carlsbad, CA). The cell nuclei and double-stranded DNA were stained with 4′, 6-diamidine-2′-phenylindole dihydrochloride (DAPI). Fluorescence images were acquired using a Zeiss inverted LSM 700 confocal microscope (Carl Zeiss, GmbH, Oberkochen, Germany).

### **Microarray analysis**

Gene Expression Microarray (Agilent Technologies) was performed at Hokkaido System Science Co. Briefly, after confirming the quality of the RNA samples with the Agilent 2100 Bioanalyzer (G2940CA), microarray analysis 100 ng of total RNA was amplified and labeled using the Agilent Low Input Quick Amp Labeling Kit, One-Color (5190-2305), and labeled RNA was hybridized to Agilent SurePrint G3 Human Gene Expression 8x60K v2 Microarrays (G4851A). Agilent Feature Extraction Image Analysis Software (Version 10.7.3) was used to extract data from raw microarray image files. Data visualization and analysis were performed using GeneSpring GX (Version 11.0) software.

### **DNA isolation and Short Tandem Repeat analysis**

The patient’s genomic DNA was isolated from PBMCs and PBMC-hiPSCs using a QIAamp® DNA mini kit (Qiagen, Valencia, CA) according to the manufacturer’s protocol. Genomic DNA was used for PCR with Powerplex 16 system (Promega Corporation, Madison, WI) and analyzed by ABI PRISM 3100 Genetic analyzer and Gene Mapper v3.5 (Applied Biosystems)

## Results

### **Reprogramming efficiencies**

**Figure 2.**
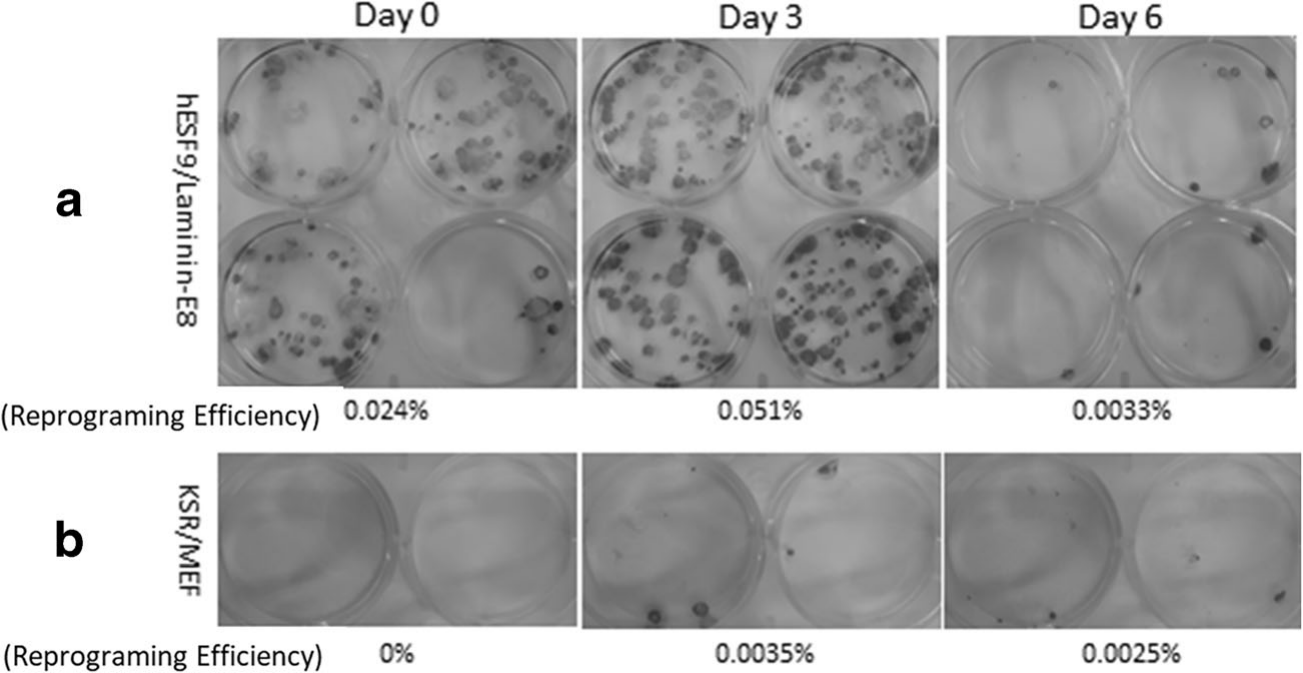
ALP-positive colonies and number of colonies after culture with IL-2 supplemented RD6F for 0, 3, and 6 d. (A) Images of ALP staining on Day 25 after transfection with SeVdp(KOSM)302L under serum-free culture conditions, displaying reprograming efficiencies of 0.024% in 0-d primary culture, 0.051% in 3-d primary culture, and 0.0033% in 6-d primary culture. (B) Images of ALP staining of control, conducted by KSR and MEF feeder co-culture under serum-supplemented conditions, displaying efficiencies of 0% in 0-d primary culture, 0.0035% in 3-d primary culture. Hamada et al.

The obtained PBMCs on Days 0, 3, and 6 with interleukin-2 (IL-2) supplemented RD6F were used for hiPSC cell reprogramming with the SeVdp(KOSM)302L vector at an MOI of 6, using Laminin-E8 under completely feeder-free and serum-free culture conditions. After 25 d, we stained each well with an ALP staining kit (Nichirei) (Fig. 2). The reprograming efficiency was calculated as ALP-positive colonies/total number of transfected cells × 100 (%). Under serum-free culture conditions, the efficiency values were recorded as 0.024% in 0-d culture, 0.051% in 3-d culture, and 0.0033% in 6-d culture (Fig. 2*A*). Under serum-supplemented condition, the values were 0% in 0-d culture, 0.0035% in 3-d culture, and 0.0025% in 6-d culture (Fig. 2*B*). Under serum-free conditions, the reprograming efficiency was 0.25–0.31% (DPC) and 0.008–0.1% (PBMC); under serum-supplemented conditions, these values were 0.30–0.44% (DPC) and 0.01–0.19% (PBMC), respectively. To ensure future applications and technical ease of handling, subsequent induction of PBMC-hiPSCs was performed on 6-d primary culture.

### **Generation of integration-free PBMC-hiPSCs**

**Figure 3.**
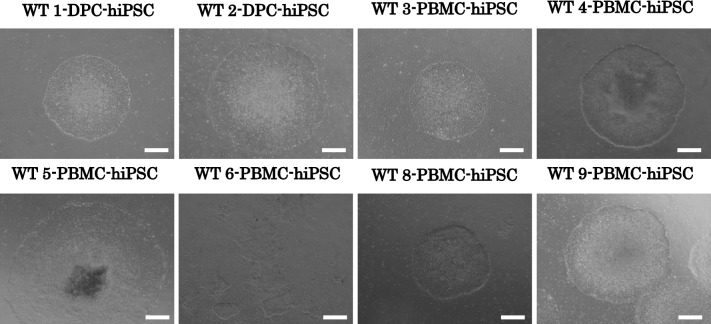
Phase contrast of DPC-hiPSCs and PBMC-hiPSCs. Phase contrast images of iPSCs derived from DPC or PBMC, WT1-DPC-iPSC clone25 at passage 40, WT2-DPC-iPSC clone6 at passage 26, WT3-PBMC-iPSC clone9 at passage 10, WT4-PBMC-iPSC clone1 at passage 10, WT5-PBMC-iPSC clone7 at passage 40, WT6-PBMC-iPSC clone3 at passage 10, WT7-PBMC-iPSC clone2 at passage 4, WT8-PBMC-iPSC clone9 at passage 5, and WT9-PBMC-iPSC clone5 at passage 16. Each hiPSC showed smooth edges and a high cell ratio in small cells. *Bars* shown in each figure are 500 μm. Hamada et al.

The schedule for PBMC-hiPSC induction is summarized in Fig. 1. At 14 d post-transfection of SeVdp(KOSM)302L into PBMC, small ESC-like colonies emerged (Fig. 1*b*, *c*), which grew to 2–3 mm, at which point several colonies were selected and mechanically passaged (Fig. 3). Each colony has a smooth edge and numerous tightly packed cells characterized by large nuclei and little cytoplasm.

### **Characterization of hiPSCs**

**Figure 4.**
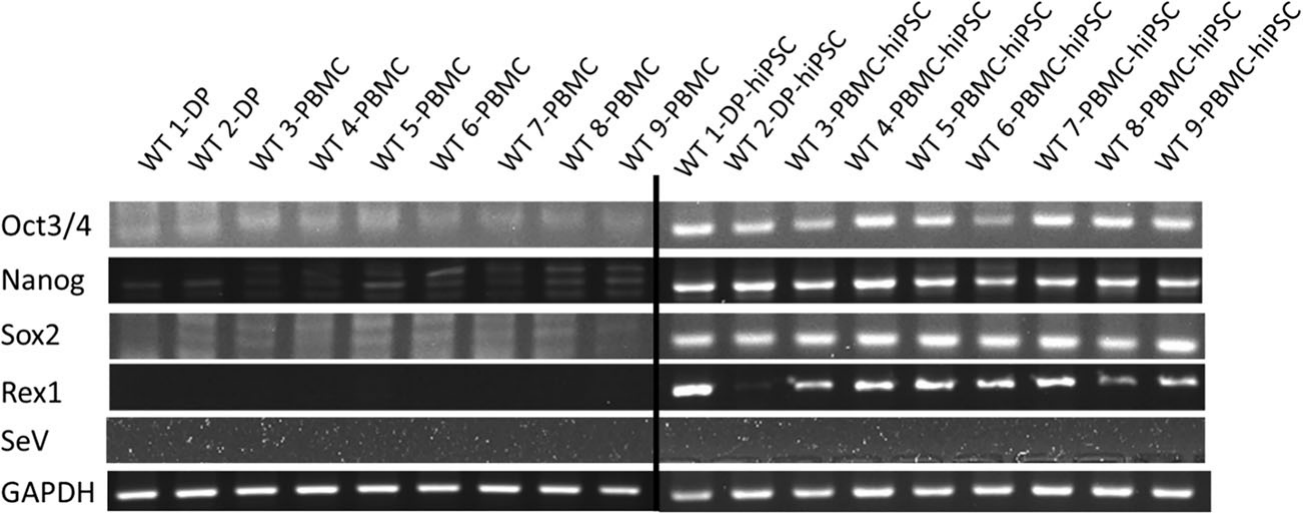
RT-qPCR. Gene expression of pluripotent markers by RT-qPCR before and after hiPSC induction of each cell line. Although *Oct3/4* was detected before reprogramming, *Nanog*, *Sox2*, and *Rex1* were expressed after reprogramming. SeVdp was not detected under all conditions. Hamada et al.

**Figure 5.**
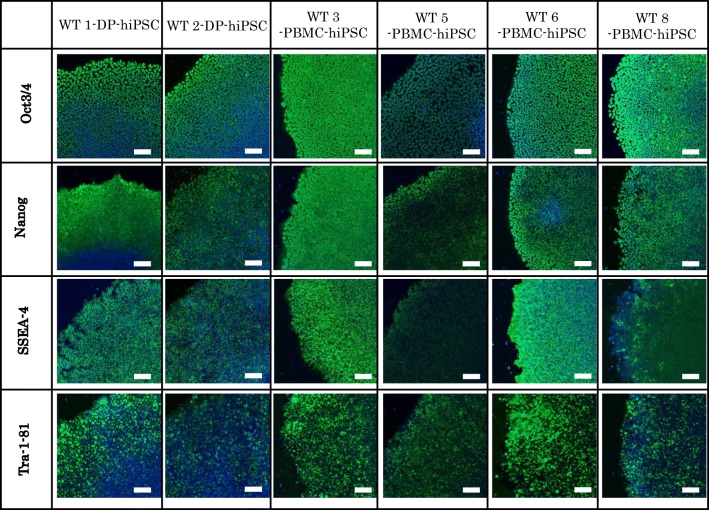
ICC (pluripotency). Immunocytochemistry of pluripotency marker proteins WT-iPSCs. WT-iPSCs were fixed and reacted with antibodies (Oct4, Nanog, SSEA-4, and Tra-1-81). Binding of these antibodies was visualized with Alexa Fluor® 488-conjugated secondary antibodies (*green*). Nuclei were stained with DAPI (*blue*). *Scale bars* represent 100 μm. Hamada et al.

**Figure 6.**
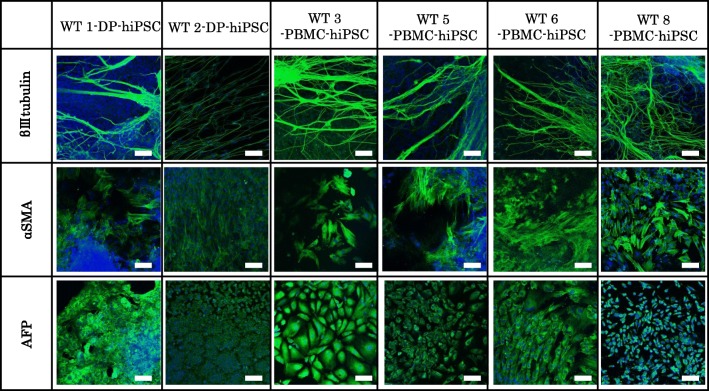
ICC (*in vitro* differentiation ability). Immunofluorescence staining of differentiation markers after 3-wk differentiation using embryoid body formation *in vitro* in each WT-hiPSCs. Immunocytochemistry of βIII-tubulin (ectoderm), α-smooth muscle actin (α-SMA, mesoderm), and α-fetoprotein (AFP, endoderm) are shown. Binding of these antibodies was visualized with Alexa Fluor® 488-conjugated secondary antibodies (green). Nuclei were stained with DAPI. *Bar* indicates 100 μm. Hamada et al.

**Figure 7.**
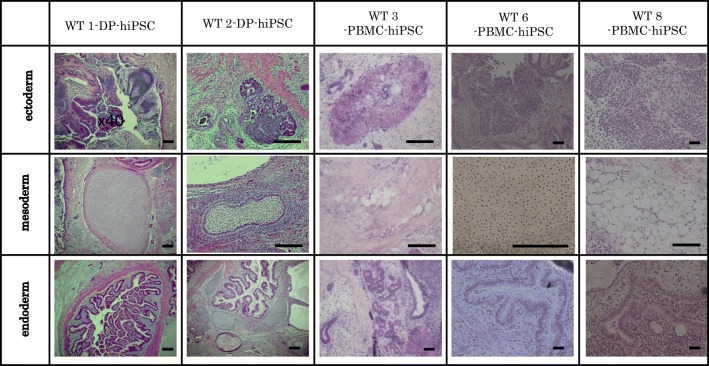
H-E staining (*in vivo* differentiation ability). WT-hiPSCs formed teratomas in SCID mice (CB17/Icr-*Prkdc scid*/CrlCrlj). Histological analysis with H-E staining demonstrated that those tumors contained various tissues, including gut-like epithelial tissues (endoderm), cartilage (mesoderm), and neural tissues (ectoderm), and were confirmed as teratomas. *Scale bars* represent 200 μm. Hamada et al.

**Figure 8.**
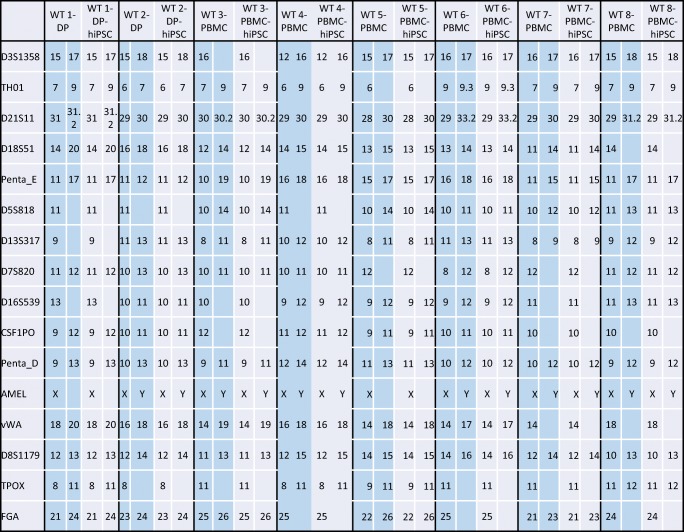
STR (short tandem repeat) analysis. STR analysis showing a match (16 loci out of 16) to verify that hiPSCs are derived from the same individual. Hamada et al.

The selected hiPSCs expressed pluripotent markers detected by RT-PCR (*Oct*, *Nanog*, *Sox2*, *Rex1*) (Fig. 4) and immunofluorescence staining (Oct, Nanog, Tra1-60, SSEA4) (Fig. 5). To determine the differentiation ability of the cells *in vitro*, embryoid body assay was performed. Immunocytochemistry detected cells positive for βIII-tubulin, smooth muscle actin, and α-fetoprotein, which are markers for ectoderm, mesoderm, and endoderm, respectively (Fig. 6). To test pluripotency *in vivo*, teratoma formation assay was performed. Histological examination showed that the teratomas formed in SCID mouse contained various tissues, including gut-like epithelial tissues (endoderm), cartilage (mesoderm), and neural tissues (ectoderm) (Fig. 7). Short Tandem Repeat (STR) results showed that PBMC-hiPSCs were derived exactly from each PBMC (Fig. 8).

### **Microarray analysis**

**Figure 9.**
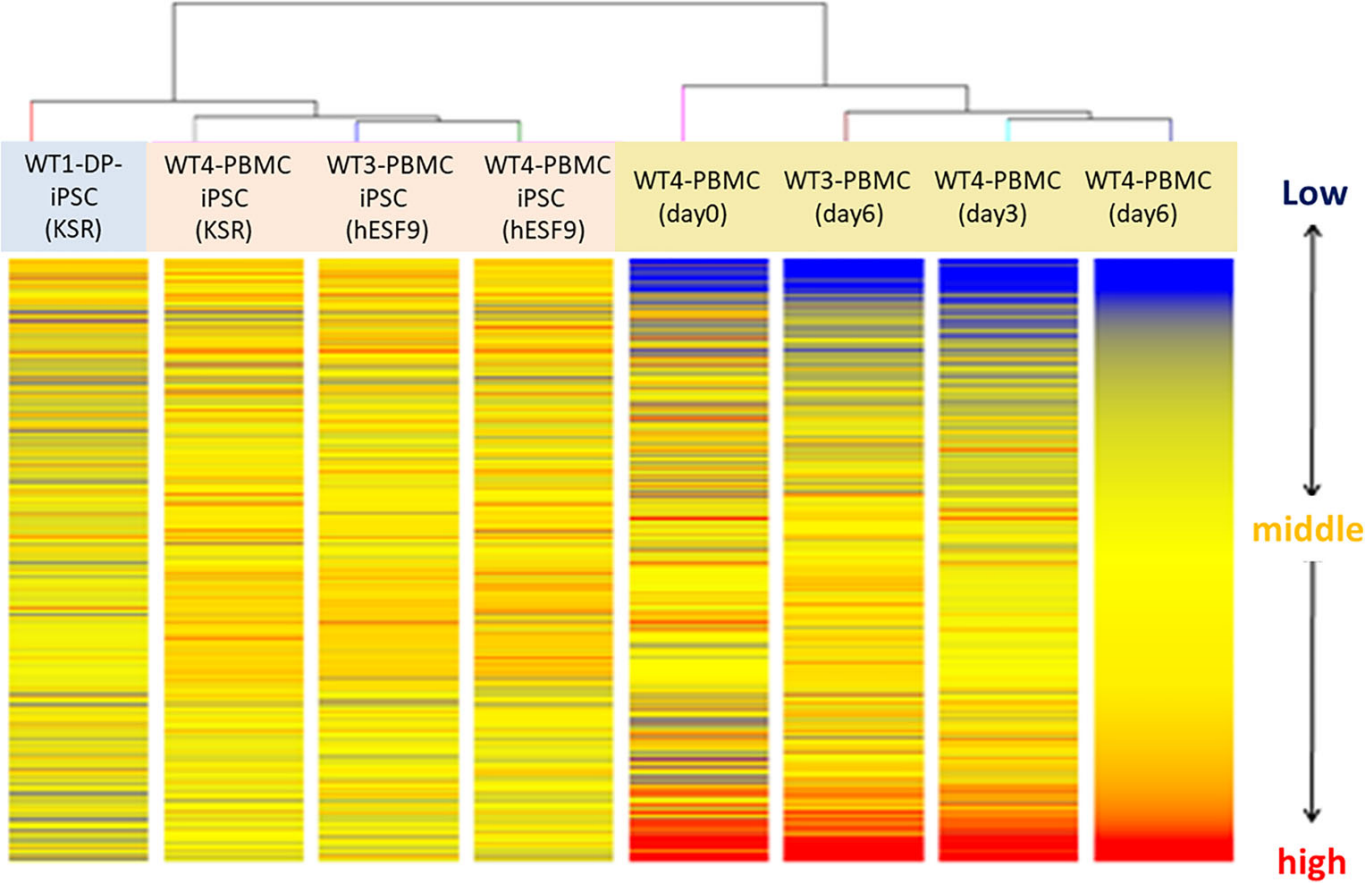
Microarray analysis. A comparison of the global gene expression patterns between PBMCs during primary culture on Days 0–6 and hiPSCs. Clustering analysis was performed based on WT4-PBMC (cultured for 6 d). Blue yellow and red indicate low, middle, and high expression level, respectively. Hamada et al.

Cluster analysis was performed on PBMCs cultured for 0, 3, and 6 d with IL-2-supplemented RD6F serum-free medium: PBMC-hiPSCs induced under serum-free condition (hESF9), PBMC-hiPSCs induced under serum-supplemented conditions (hKSR), and DPC-hiPSCs induced under serum-free conditions (hESF9). The gene expression patterns in PBMC group and PBMC-hiPSCs group were similar; PBMC-hiPSCs exhibited a very different expression pattern from that of PBMCs before induction, and PBMC-hiPSCs showed a similar expression pattern as DPC-hiPSCs (Fig. 9).

Next, the gene expressions of PBMC-hiPSC isolated under different culture conditions were analyzed. WT4-PBMC iPSC induced in hESF9 displayed expression pattern more similar to that of WT3-PBMC hiPSC induced in hESF9 than that of WT4-PBMC hiPSC induced in serum-supplemented condition (Fig. 9). In other words, the gene expression pattern of PBMC-hiPSCs established under serum-free conditions is more similar to that of PBMC-hiPSCs established under serum-free conditions from another individual than that of those established under serum-supplemented conditions from the same individual. In summary, differences in culture conditions are concluded to have a greater effect on gene expression differences than differences in individuals from whom the cells were sourced.

## Discussion

We previously reported induction of hiPSCs from fibroblasts derived from gingiva or DPCs derived from extracted teeth (Yamasaki *et al.*^2016^; Yamasaki *et al.*^2014^). However, the extraction of skin, mucous, or dental pulp involves surgical procedure, and, in particular, target patients may not have any teeth for dental pulp extraction. Since our eventual goal is to establish a general method to induce hiPSCs from genetic disorder patients, the donor population must be bigger. Hence, it is important that we improve hiPSC induction procedure by reducing biopsy invasiveness. However, the induction efficiency of hiPSCs from PBMCs is extremely low compared to that of skin fibroblasts using conventional induction methods (Staerk *et al.*^2010^). We induced PBMC-hiPSCs multiple times using conventional methods but failed to obtain positive results. The SeVdp(KOSM)302L non-integrating viral vector used in this report encodes all four reprograming factors (Klf4, Oct3/4, Sox2, c-Myc) to achieve efficient factor expression. By incorporating this expression vector into a reprogramming protocol that included serum-free and feeder-free culture conditions, we induced and successfully recovered hiPSCs from PBMCs. The efficiency of hiPSC induction from PBMCs was approximately one half to one third of that from DPCs, but was sufficiently high for routine use. Induction of PBMC-hiPSCs has been reported using other viral expression vectors in combination with feeder cells in serum-supplemented medium. Brown *et al.* (2010) reported an induction efficiency of hiPSCs using retrovirus of 0.01%, and Loh *et al.* (2009) reported an induction efficiency of 0.0008–0.001% using lentiviruses. Nakagawa *et al.* (2014) used an episomal vector under serum-free culture conditions and reported an induction efficiency of 0.001–0.011%. Kishino *et al.* (2014) reported PBMC-hiPSC induction under serum-free conditions using Sendai virus vectors carrying one gene per vector, with an induction efficiency of 0.005%. Trokovic *et al.* (2014), using serum-free conditions, also reported an induction efficiency of 0.005%. In each of these cases, the efficiency of inducing PBMC-hiPSCs was extremely low as compared to the results of the present study in which induction efficiency was 0.008–0.1% using SeVdp(KOSM)302L carrying four genes in one vector.

Previously, we examined a variety of extracellular matrix molecules (ECMs) including fibronectin, type I collagen, and gelatin for their suitability for DPC-hiPSC induction and confirmed that fibronectin was the most suitable (Yamasaki *et al.*^2014^). Prior to this study, we re-examined PBMC-hiPSC induction by adding Laminin-E8 to previously characterized ECMs under serum-free condition. We successfully generated hiPSCs on all ECMs; in particular, colonies appeared more rapidly and the induction efficiency was higher in the presence of Laminin-E8 (supplement data). While Matrigel is also widely used in hiPSC induction protocols as ECM under serum-free medium, its use is potentially problematic because Matrigel contains many undefined factors that may affect cell growth and differentiation. Laminin is the main component of Matrigel and Laminin-E8 is the minimum fragment conferring integrin-binding activity. Miyazaki have reported that Laminin-E8 has the potential to promote the survival of hiPSCs by robust adhesion via integrins and phosphorylation of AKT, ERK1/2, and FAK, which are highly phosphorylated in human pluripotent stem cells (Miyazaki *et al.*^2012^). Thus, Laminin-E8 was adopted as ECM for our system.

Prior to inducing hiPSCs from blood cells, granulocyte macrophage colony-stimulating factor (GCSF) was added to stimulate the proliferation of specific T cells, B cells, and granulocytes using cytokines, such as stem cell factor and IL-3 (Loh, *et al.*, 2009; Seki, *et al.*, 2010). Based on a culture method similar to that used to activate lymphocytes for cell therapy performed on patients with oral cancer in our department, natural killer (NK) cells and cytotoxic T cells with strong cytotoxic activity were stimulated to proliferate with IL-2 in this study. By inducing hiPSCs from these cells, we also aimed to differentiate hiPSCs into cells with high cytotoxic activity for cell therapy. When inducing hiPSCs from PBMCs in RD6F medium containing IL-2, considerable individual differences were observed in induction efficiencies. This tendency was marked in reprogrammed cells cultured on MEF feeder cells. This suggests that lymphocytes with high cytotoxic activity attacked non-autologous mouse feeder cells or self-lymphocytes transfected with Sendai virus, resulting in reduced induction efficiency. Supporting this hypothesis, differences in marker gene expression determined by DNA microarray between individuals with the highest and lowest induction efficiencies revealed that individuals with lower induction showed higher expression of cytotoxic activity-related genes. Although hiPSC induction was carried out using activated lymphocytes with high cytotoxic activity with the goal of future cell therapy, when aiming to induce hiPSCs from PBMCs with high efficiency, induction should be performed immediately after isolation without IL-2 stimulation. In addition, the potential problems associated with isolating hiPSC from PBMC on mouse feeder cells can be avoided by using our feeder-free and serum-free culture method. Finally, our reprogramming protocol that does not use an integrating viral vector is preferable for generating hiPSCs for clinical applications.

Our defined culture system is advantageous for studying the regulation of cell differentiation and for generating and maintaining pluripotent cells for clinical applications. The combination of this system and the generation of patient-derived iPSCs will contribute to providing us with new knowledge to overcome various disorders. Furthermore, disease-relevant cells differentiated from patient-derived iPSCs will become a powerful tool for investigating pathogenesis *in vitro* and accelerate the development of effective therapies.

## Conclusions

Here, we described the successful generation of PBMC-hiPSCs under serum- and feeder-free conditions. These PBMC-hiPSCs will be helpful for clinical applications and drug discovery. The ability to establish hiPSCs efficiently from PBMCs with SeVdp(KOSM)302L will enable the generation of normal and disease-specific hiPSCs from healthy people and patients with rare genetic disorders, thus facilitating studies of the mechanisms of action disease onset.
